# Rapid and Simultaneous Quantification of Six Aristolochic Acids and Two Lignans in Asari Radix et Rhizoma Using Ultra-Performance Liquid Chromatography-Triple Quadrupole Tandem Mass Spectrometry

**DOI:** 10.1155/2022/5269545

**Published:** 2022-09-10

**Authors:** Hanze Liu, Xuemei Cheng, Huida Guan, Changhong Wang

**Affiliations:** Institute of Chinese Materia Medica, Shanghai University of Traditional Chinese Medicine, the MOE Key Laboratory for Standardization of Chinese Medicines, Shanghai Key Laboratory of Compound Chinese Medicines, Shanghai R&D Centre for Standardization of Chinese Medicines, the SATCM Key Laboratory for New Resources and Quality Evaluation of Chinese Medicines, 1200 Cailun Road, Shanghai 201203, China

## Abstract

Asari Radix et Rhizoma (AR) is a widely-used Chinese herbal medicine containing multiple active lignans and rare nephrotoxic components-aristolochic acids derivatives (AAs). However, the current quality control method carried out by Chinese Pharmacopoeia has defects in trace AAs detection and insufficient marker ingredients, which is unable to comprehensively evaluate the efficacy and safety of AR. To improve the quality control method of AR, a rapid, sensitive, and reliable chromatographic analytic method based on ultra-high-performance liquid chromatography-triple quadrupole tandem mass spectrometry (UHPLC-QqQ-MS) was established for the simultaneous analysis of multiple AAs and lignans in AR samples. Positive electrospray ionization mode with multiple reaction monitoring (MRM) was applied for the detection of the eight analytes. The method showed available linearity (*R*^2^ ≥ 0.991), the limit of quantification (2–5 ng/mL), precision (RSD <8.12%), and accuracy (89.78–112.16%). A total of 6 AAs and 2 lignans were quantified for their content in 15 AR samples. The content of AA-IVa, AA-VIIa, and aristololactam I (AL-I) was much higher than the AA-I controlled by pharmacopoeia. Considering the potential toxicity of AAs, AA-IVa, AA-VIIa, and AL-I should also be controlled in AR. A considerable amount of active sesamin was detected in AR, suggesting that it could be added as a quality marker for the quality control of AR. The newly developed analytical method could be applied for the fast evaluation of toxic AA's content and quality during quality control of AR or preparations containing AR.

## 1. Introduction

Asari Radix et Rhizoma (AR) is the roots and rhizomes of *Asarum heterotropoides* Fr. Schmidt var. *mandshuricum* (Maxim.) Kitag, *Asarum sieboldii* Miq. var. *seoulense* Nakai, or *Asarum sieboldii* Miq. More than 40 kinds of traditional Chinese patent medicines and prescriptions have used AR for the treatment of cold, headache, toothache, runny nose, rhinorrhea, rheumatism, and cough with phlegm retention. In the existing quality control standards, asarinin and aristolochic acid I were approved as indicating ingredients of AR [1] due to their strong bioactivities [2] and potential nephrotoxicity [3], separately.

As a plant of the Aristolochiaceae, the possible risk of aristolochic acid nephropathy caused by herbal medicine will naturally arouse vigilance. Aristolochic acid derivatives (AAs) are the culprits of aristolochic acid nephropathy reported since the late 20th century [4]. Plants containing AAs such as *Aristolochia debilis*, *Aristolochia manshuriensis*, *Aristolochiae fangchi*, and *Aristolochia contorta* were banned and restricted for their medicinal use by the British Committee on the Safety of Medication and the U.S. Food and Drug Administration successively [5]. The safety issue of Chinese medicine containing AAs has become the focus of attention in domestic and foreign medical circles at the beginning of the 21st century. A recent study showed that AAs are mainly distributed in the leaves and fruits of the whole AR plant [6]. In addition, the content of AAs in some AR of nonofficial species was significantly higher than that of official species [7–10]. As a result, a variety of confusion, residue of aboveground parts, or improper processing of original plants may increase the risk of AR medication. The content of aristolochic acid (AA)-I in AR was limited to 0.001% by the Chinese pharmacopoeia in 2005. However, recent studies have shown that AAs such as AA-II, AA-III, AA-IVa, AA-VIIa, and aristololactam I (AL-I) possibly existing in AR [11, 12] are also cytotoxic to renal tubular epithelial cells as AA-I more or less [13–16]. The standards of pharmacopoeia may neglect the toxicity of AAs other than AA-I.

**Figure 1:**
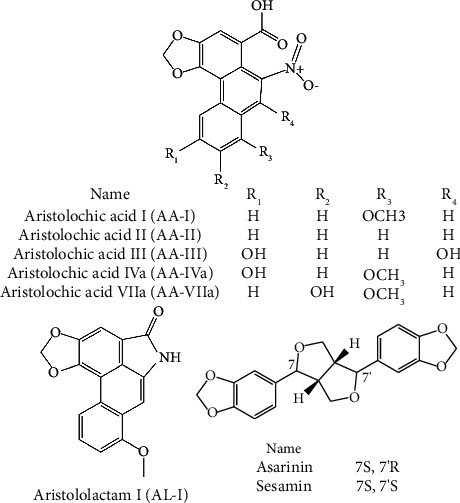
Chemical structures of analytes in this study.

Asarinin and sesamin, the major ingredients pertaining to the lignan phytochemical group in AR, have a variety of pharmacological activities consistent with the efficacy of AR, including antipyretic, antiinflammatory, and immunosuppressive effects [2, 17–23]. The latest network pharmacology research also shows that asarinin and sesamin may be the key active ingredients for AR to exert antiinflammatory and analgesic effects. They play a critical role in evaluating the quality of AR [24]. Nevertheless, only asarinin was selected as the marker ingredient for the quality control of AR [1]. The content of asarinin may not fully reflect the holistic quality of AR. More marker ingredient candidates need to be investigated for the improvement of quality control of AR. The chemical structures of lignans and AAs were shown in Figure 1.

Until now, a variety of qualitative analyses and quantitative determination of AR components have been developed, including high-performance liquid chromatography (HPLC) coupled to photodiode array detection [25–29], fluorescent detection (FLD) [12, 30, 31], and electrochemical detection [32]. These traditional analytical methods often suffer from poor sensitivity or dependence on chemical derivatization for the detection of trace AAs in AR [33]. The liquid chromatography-tandem mass spectrometry (LC-MS) method has been widely employed for the analysis of AAs in AR plants or preparations due to the improved sensitivity and high specificity [11, 28, 34, 35] that enabled in-depth progress in qualitative analysis and quantitative determination of various trace AAs in plants and products. In the Chinese pharmacopoeia, the contents of AA-I and asarinin in AR were analyzed by two separate HPLC methods [1], which resulted in low throughput. Considering the complexity of the herbal matrix, a highly selective method is necessary to make a rapid analysis of various types of ingredients available. The application of ultra-high-performance liquid chromatography-triple quadrupole tandem mass spectrometry (UHPLC-QqQ-MS) can effectively avoid the interference of overlapping peaks and give an extremely low limit of detection and quantitation, which is suitable for the simultaneous analysis of multiple compounds. MRM monitoring mode can eliminate coelution interferences and background noise, so as to improve the signal-to-noise ratio (S/N) for some analytes. The UHPLC column packed with sub-2-um particles can significantly increase the theoretical plate number that enables higher analytical efficiency [36].

Although the content of AAs in AR is negligible, consumers can still purchase AR with uncertified sources from a number of websites. Moreover, AR whole plants are habitually used in some districts, which contain higher AAs than roots and rhizomes [6]. These uncertified ARs are likely to be misidentified as or substituted for a certified AR, which may lead to a potential risk of causing aristolochic acid nephropathy. Therefore, in order to control the safety and effectiveness of AR effectively, it is urgently needed to develop a rapid and sensitive analytical method for the simultaneous determination of key quality control ingredients in AR to make up for the shortcomings of the previous quality control methods. Based on the importance of lignans and AAs in the quality control of AR, a rapid and sensitive UHPLC-QqQ-MS method was developed to simultaneously determine AA-I, AA-II, AA-III, AA-IVa, AA-VIIa, AL-I, asarinin, and sesamin in this paper.

## 2. Material and Methods

### 2.1. Chemicals and Reagents

Reference standards AA-I, AA-III, AA-IVa, and asarinin were purchased from Chengdu Aifa Biotechnology Co., Ltd., AA-II, AA-VIIa, and AL-I were purchased from Shanghai Hongyong Biotechnology Co., Ltd., sesamin was purchased from Shanghai Yuanye Biotechnology Co., Ltd. HPLC-grade methanol, acetonitrile, and formic acid were purchased from Fisher Scientific Co. (Santa Clara, CA, USA). Deionized water was purified using a Milli-Q Academic System made by Millipore Co. (Billerica, MA, USA). All other chemicals were of analytical grade.

### 2.2. Plant Materials

Samples of AR were collected from Liaoning province or purchased from Bozhou Traditional Chinese Medicine Market. Their supplementary information was listed in Table 1. These materials were authenticated as the dried roots and rhizomes of *Asarum heterotropoides* Fr. Schmidt var. *mandshuricum* (Maxim.) Kitag authenticated by Prof. Lihong Wu (Shanghai Standardization Research Center for Traditional Chinese Medicine). The voucher specimens were deposited in the specimen room of the Shanghai Standardization Research Center for Traditional Chinese Medicine.

### 2.3. Instrumentation and Chromatographic Conditions

The UHPLC-QqQ-MS analysis was performed on an Agilent 1290-UHPLC system (Agilent Technologies, California, USA) coupled with an Agilent 6410 Triple Quad liquid chromatography-tandem mass spectrometry system (Agilent Technologies Inc., Santa Clara, CA) at positive ion mode as the quantitative analysis instrument. The separation was run on the ACQUITY UPLC BEH C18 column (50 mm × 2.1 mm, id 1.7 *μ*m) at 40°C. The mobile phase consisted of water containing 0.1% formic acid (*A*) and acetonitrile (*B*) with gradient elution programmed as follows: 0–2 min, 10%–45% *B*; 2–6 min, 45%–60% *B*; 6-7 min, 95% *B*; 7-8 min, 10% *B*. The flow rate was kept at 0.4 mL/min, and 5 *µ*L of standard and sample solution were injected in each run.

ESI-MS/MS conditions such as gas pressure 350°C, gas flow 12 L/min, capillary 4000 V, nebulizer pressure 45 psi, and the optimized MS analytical parameters of eight compounds were shown in Table 2. The optimized MRM parameters mainly relied on the absolute response of the selected ion pair by changing the fragmentor and collision energies. The corresponding MRM chromatographic peaks of sesamin and asarinin were identified by the injection of a single standard separately.

### 2.4. Preparation of Standard Solutions

Eight standard stock solutions (100 *μ*g/mL for AAs, 1000 *μ*g/mL for asarinin and sesamin) were independently prepared by dissolving in an appropriate amount of methanol and stored at −20°C. An appropriate amount of standard stock solutions were mixed and diluted by methanol to get a mixed standard stock solution at a final concentration of 10 *μ*g/mL for AAs and 100 *μ*g/mL for asarinin and sesamin. The working solutions were prepared by the dilution of standard stock solutions to obtain the required concentrations for the method validation (accuracy and precision, limit of detection and quantification). The calibration standard solutions of eight concentration levels (5, 10, 20, 100, 200, 500, 750, and 1000 ng/mL for each AAs standard and 50, 100, 200, 1000, 2000, 5000, 7500, and 10000 ng/mL for asarinin and sesamin) were prepared by diluting the above stock solutions. All these solutions were stored at 4°C in a refrigerator.

### 2.5. Sample Preparation

The 0.5 g powdered crude drug samples (65 meshes, 0.230 mm) were accurately weighed and extracted by ultrasonic (500 W, 40 kHz) with 15 mL 70% methanol for 45 min. The extract was cooled down to room temperature and compensated by weight with 70% methanol. The solution was centrifuged at 3000 rpm for 5 min, then the supernatants were filtered with a 0.22 *μ*m filter membrane, with 1 mL initially filtered filtrates discarded, and an aliquot of each 5 *µ*L was injected into the UHPLC system for analysis.

### 2.6. Validation of the Method

#### 2.6.1. Calibration Curves

Calibration curves were prepared with the working solutions as described in Section 2.4 for each validation run using external standard calibrations for eight analytes with a weighted least square power regression and then constructed by plotting the peak area versus the concentration of each analyte.

#### 2.6.2. Limit of Detection and of Quantitation

The stock solutions of eight reference compounds were diluted to a range of 2–50 ng/mL. The injection volume was 5 *µ*L. The LOD was defined as the concentration for which the signal-to-noise (S/N) of 10 was obtained.

#### 2.6.3. Precision, Accuracy, Repeatability, Stability, and Recovery

The precisions were evaluated by the analysis of six injections of working solutions at four concentrations, that is, 5, 20, 100, and 750 ng/mL for each AAs standard and 50, 200, 1000, and 7500 ng/mL for asarinin and sesamin. The LLOQ of the assay was quantitated using accuracy within 20% bias of the nominal concentration and relative standard deviation not exceeding 20%. Six different sample solutions prepared from the same sample were analyzed to confirm the repeatability of the developed method. The stability of the sample was tested by injecting the same amount of sample preparation at 0, 2, 4, 6, 8, 10, 12, 16, 24, and 48 h stored in a sample plate at 10°C. The peak area of the stability samples was substituted into the calibration curve to calculate the concentration of the analytes. The RSD value of the concentration of each analyte was then calculated to obtain the stability and repeatability results. The recovery was used to evaluate the accuracy of the method. For the recovery testing, approximately 0.5 g of the fine powder of sample no. 6 was accurately weighted, then accurate amounts of mixed standards (about 50%, 100%, 150% of the amount in sample no. 6, *n* = 3) were added to the herb. At last, the herb was extracted and analyzed as described in Section 2.5. The recovery value was calculated by the following equation: recovery (%) = (detected amount - original amount)/spiked amount × 100%.

#### 2.6.4. Data Analysis

All calibration and quantitation data were processed with Agilent Technologies Mass Hunter Workstation Quantitative Analysis software version B.05.00. The experimental data were expressed as the mean ± SD.

The significance analysis was processed by using GraphPad Prism 5 software. Statistical analysis was performed by using ANOVA with *p*=0.05 as the minimum level of significance.

## 3. Results and Discussion

### 3.1. Optimization of MS/MS Condition

For optimization of MS conditions, the full-scan MS method was used to examine the target analytes in positive ionization mode. All the compounds were then determined, respectively, in direct infusion mode to optimize a proper transition for the MS/MS detection. (M+H)^+^, (M + NH_4_)^+^, and (M + H-H_2_O)^+^ were the basic protonated ions for eight analytes under positive ion mode. Base peak with the highest response was selected as precursor ion for AA-I, AA-II, AA-III, AA-VIIa, AL-I, asarinin and sesamin, except for AA-Iva selected (M + H-NO_2_)^+^ as precursor ion to distinguish from AA-VIIa. Then, the conditions of multiple reaction monitoring (MRM) determination, including fragmentor, collision energy, and cell accelerating voltage, were optimized according to the highest sensitivity and specific ion pairs. The MRM transitions and parameters of sesquiterpene lactone compounds are shown in Table 2.

### 3.2. Method Validation of UHPLC-MS/MS

#### 3.2.1. Linearity, LOQ, Repeatability, and Stability

The eight-point calibration curves of eight analytes (AA-VIIa, AA-I, AA-II, AA-III, AA-IVa, AL-I, sesamin, and asarinin) showed available linearity ranging from 5 to 1000 ng/mL (sesamin and asarinin ranging from 50 to 10000 ng/mL) by analyzing standard working solutions at eight concentrations, and the typical equations of the calibration curves are shown in Table 3. All standard curves offered the correlation coefficient (*R*^2^) ranging from 0.9912 to 0.9989 for eight analytes within the linear ranges, indicating its feasibility for quantification.

The LOQ of each analyte (AA-VIIa, AA-I, AA-II, AA-III, AA-IVa, AL-I, sesamin, asarinin) was 2, 2, 5, 5, 2, 2, 50, and 50 ng/mL, respectively, demonstrating the good sensitivity of the established method.

Repeatability and sample stability were evaluated by the relative standard deviation (RSD) values presented in Table 4. The experimental operation was repeatable for six analytes (AA-II and AA-III were not detected in samples) in six independently prepared samples with an RSD of less than 5.45%, and six investigated compounds in a newly prepared AR sample were stable when kept in the autosampler (10°C) for 48h with an RSD of less than 6.52%.

#### 3.2.2. Accuracy and Precision

The precision of eight analytes (AA-VIIa, AA-I, AA-II, AA-III, AA-IVa, AL-I, sesamin, and asarinin) at four levels was within 8.12%, and the accuracy of the eight analytes ranged from 89.78% to 112.16%, which were within the acceptable limits. All data of precision and accuracy were summarized in Table 4. The results demonstrated that the stability-indicating method was reliable and accurate.

#### 3.2.3. Recovery

As summarized in Table 5, the extraction recoveries of the six analytes in samples at three evaluated concentrations were within the range of 81.58%–109.73%, indicating good accuracy for eight analytes.

### 3.3. Determination of Analytes in Crude Drugs

**Figure 2:**
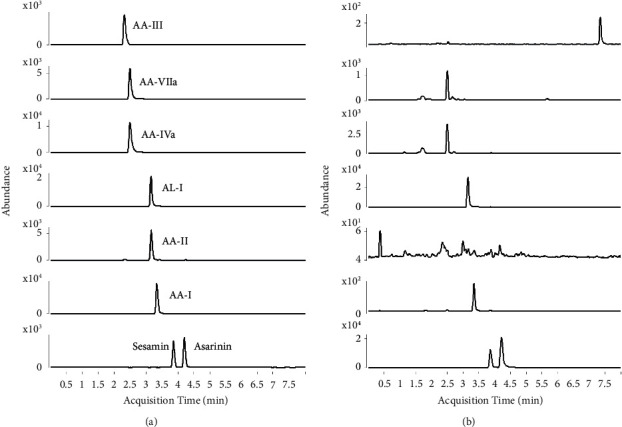
Liquid chromatogram of analytes in MRM mode. (a) MRM of mixed standards and (b) MRM of AR extract sample.

**Figure 3:**
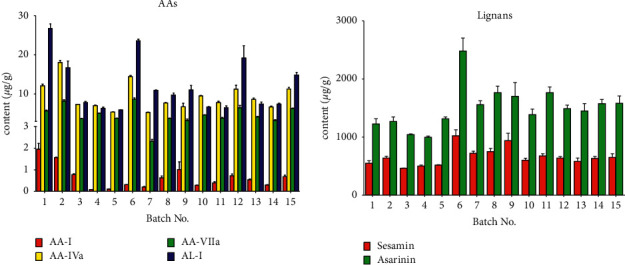
Content of AAs and lignans in 15 batches of crude drugs samples (mean ± SD, *n* = 3).

Using the developed UHPLC-QqQ-MS method, quantitation of AA-I, AA-IVa, AA-VIIa, AL-I, asarinin, and sesamin in 15 batches of AR samples was carried out. The typical MRM chromatograms of standard solution and sample solution were shown in Figure 2, in which the retention time of AA-VIIa, AA-I, AA-II, AA-III, AA-IVa, AL-I, asarinin, and sesamin were 2.477 min, 3.336 min, 3.155 min, 2.306 min, 2.479 min, 3.141 min, 4.21 min, and 3.87 min, respectively. As shown in Figure 3, the quantitative determination results showed that the average content of AA-VIIa, AA-I, AA-IVa, AL-I, sesamin, and asarinin in 15 batches of AR were 4.86, 0.64, 9.28, 12.06, 659.00, and 1507.04 *μ*g/g, respectively. AA-II and AA-III were not detected in any sample. The total content of all AAs detected was 26.83 *μ*g/g on average. All AR samples meet the standards of marker ingredient AA-I (≤0.001%, 10 *μ*g/g) and asarinin (≥0.05%, 500 *μ*g/g) in the Chinese pharmacopoeia [1]. Among 15 herbal samples, the highest content of AA-I is 1.96 *μ*g/g, which is far below the upper limit of 10 *μ*g/g. The lowest content of asarinin is 995.88 *μ*g/g, which is about twice the folds of the lower limit. Among other AAs derivatives, a considerable amount of AA-VIIa, AA-IVa, and AL-I were detected in all AR samples and showed a higher content than AA-I (0.64 *μ*g/g), with an average content of 4.86, 9.28, and 12.06 *μ*g/g, respectively. At the same time, a considerable amount of sesamin with an average content of 659.00 *μ*g/g was found. The obtained results provided a reference for the profile of the lignans and AAs presented in AR, which would be beneficial for quality control of AR in the future.

For the analysis of AAs, researchers focus on developing FLD [37] or MS [30] detectors with a precolumn derivatization method to improve the detection sensitivity (such as the limit for detection for AA-I can reach 0.02–0.73 ng/mL). When considering the universality of analysis methods for drugs, fewer pretreatment steps and shorter analysis time should be taken into account. The current analytical method keeps the limit of quantification of six AAs at a relatively low level (2–5 ng/mL), which not only simplifies the pretreatment steps but also has adequate sensitivity to meet the demands of AR quality control. In addition, the method can quantitatively determine the active ingredients asarinin and sesamin simultaneously, which are the key quality control ingredients in AR. In comparison to an analysis of the comprehensive characterization of 22 AR components by using ultra-high-performance liquid chromatography-time of flight/mass spectrometry (UHPLC-QTOF/MS) [10], the current method aims at most concerning toxic AAs and representative active lignans in quality control. The analysis time for a single sample was shortened from 25 minutes to 8 minutes, which greatly improved the analysis efficiency.

With regards to the toxicity of AAs derivatives, early reports have believed that AA-I and AA-II are the major toxic components among AAs [38–40]. According to previous studies, AA-I has shown the most toxicity among AAs derivatives to renal epithelial cell lines *in vitro* [40]. AA-IVa is less toxic to P388 cell lines and *Salmonella* strains and nontoxic to LLC-PK1 cells [40]. AL-I is toxic to P388 and human epidermoid cancer cells [41], yet proved to be nontoxic in LLC-PK1 cells [40]. *In vivo*, AA-I showed the strongest nephrotoxicity in mice; AA-II has mild nephrotoxicity; AA-IVa and AL-I do not cause blood chemistry or tissue abnormalities of the kidneys, as indicated by the academic changes [42]. However, a few more studies found that numerous AAs have similar toxic effects to AA-I. For example, AL-I is the nitro reduction product of AA-I. Mutual transformation may happen during the extraction process. Aristololactam derivatives have also been reported to have in vitro cytotoxicity [43–45]. Although the cell damage mechanism of AL-I is different from AA-I, it can still cause the increase of extracellular matrix components in vitro the same as AA-I [14, 15]. Moreover, other AAs derivatives such as 7-methoxy-aristololactam IV and aristololactam IVa exhibited similar or even higher cytotoxicity than AA-I in MTT and lactate dehydrogenase leakage assays [13]. Related structure-activity relationship studies have shown that in addition to the nitro group as a structural requirement for AAs-mediated cytotoxicity, the presence of methoxy and hydroxyl also plays an important role [40], emphasizing the potential nephrotoxins of AAs derivatives other than AA-I that may exist in AR. In summary, there is still controversy over the relative toxicity of AAs, so it is necessary to be wary of the toxicity caused by these compounds.

From the perspective of quality control, safety and effectiveness are the key factors in controlling the quality of medicinal materials. In this study, the potential toxic AAs were detected in all 15 AR samples, among which marker ingredient AA-I accounted for only 2.38% of the total AAs on average. In order to ensure the safety of AR more accurately, the limitation of the total number of AAs is a proposal worth considering. More nephrotoxicity-related evaluation studies are still required to clarify the specific upper limit of AAs. In addition, a considerable amount of sesamin was also found in each sample. In view of the similar pharmacological activities of sesamin and the existing quality marker asarinin, it is recommended to add sesamin as the quality marker in the following quality standard of AR.

## 4. Conclusions

In conclusion, a rapid and sensitive UHPLC-QqQ-MS method was established for the simultaneous quantification of six aristolochic acids and two lignans in AR, which was validated with good accuracy and precision. AA-I, AA-IVa, AA-VIIa, AL-I, asarinin, and sesamin were detected in all 15 AR samples by the developed method, and their content was clarified. The content of AA-IVa, AA-VIIa, and AL-I was much higher than AA-I in all AR samples, indicating that AA-IVa, AA-VIIa, and AL-I should be limited together to a certain extent for the safety use of AR. Active ingredient sesamin is also recommended to be added as a quality marker for the improvement of quality control of AR. The newly developed analytical method could be applied for the fast evaluation of toxic AAs content and quality during quality control of AR commercial medicinal materials or the preparations of AR contained.

## Figures and Tables

**Table 1:** List of batch no., collection date, provenance, and acquisition manner for plant samples investigated.

No.	Batch no.	Collection date	Provenance	Acquisition manner
1	20191003	2019.10.3	Xinbin county, Liaoning	Gathered
2	20191004–1	2019.10.4	Xinbin county, Liaoning	Gathered
3	20191004–2	2019.10.4	Xinbin county, Liaoning	Gathered
4	20191004–3	2019.10.4	Xinbin county, Liaoning	Gathered
5	20191004–4	2019.10.4	Xinbin county, Liaoning	Gathered
6	20200111	2020.1.11	Xinbin county, Liaoning	Purchased
7	20200407–2	2020.4.7	Huanren Manchu autonomous county, Liaoning	Purchased
8	20200407–1	2020.4.7	Huoshan county, Anhui	Purchased
9	20200407–6	2020.4.7	Fengcheng city, Liaoning	Purchased
10	20200407–5	2020.4.7	Baishan city, Jilin	Purchased
11	20200407–7	2020.4.7	Antu county, Jilin	Purchased
12	20200407–3	2020.4.7	Xinbin county, Liaoning	Purchased
13	20200407–8	2020.4.7	Anguo county, Hebei	Purchased
14	20200409	2020.4.9	Bozhou traditional Chinese medicine market, Anhui	Purchased
15	20200407–4	2020.4.7	Dandong city, Liaoning	Purchased

**Table 2:** The MS detection parameters of analytes.

Analytes	Q1⟶Q3 (*m/z*)	Fragmentor	Collision energy (V)	Cell accelerating voltage (CAV)	Retention time (min)
AA-III	345.3⟶284.1	80	8	1	2.31
AA-VIIa	340.3⟶281.1	170	32	1	2.48
AA-IVa	312.3⟶297.2	170	27	1	2.48
AL-I	294.3⟶279.2	160	31	1	3.14
AA-II	329.2⟶268.3	80	8	1	3.16
AA-I	359.3⟶298.2	90	10	1	3.34
Sesamin	337.3⟶135.1	130	30	3	3.87
Asarinin	337.3⟶135.1	130	30	3	4.21

**Table 3:** The representative calibration curve, linear range, LOQs, stability, and repeatability of analytes (*n* = 3).

Compounds	Calibration curve	*R * ^2^	Linear range (ng/mL)	LOQ (ng/mL)	Stability (%)	Repeatability (%)
AA-III	*y* = 20.0228^*∗*^*x* ^ 0.8467	0.9981	5–1000	5	n.d.	n.d.
AA-VIIa	*y* = 17.0538^*∗*^*x* ^ 0.9483	0.9989	5–1000	2	1.18	5.68
AA-IVa	*y* = 73.1555^*∗*^*x* ^ 0.8450	0.9985	5–1000	2	1.76	3.03
AL-I	*y* = 482.1083^*∗*^*x* ^ 0.8731	0.9920	5–1000	2	2.01	2.72
AA-II	*y* = 10.4508^*∗*^*x* ^ 0.8604	0.9986	5–1000	5	n.d.	n.d.
AA-I	*y* = 43.3606^*∗*^*x* ^ 0.9850	0.9956	5–1000	2	5.45	6.52
Sesamin	*y* = 7.6321^*∗*^*x* ^ 0.9139	0.9942	50–10000	50	2.27	4.08
Asarinin	*y* = 18.8808^*∗*^*x* ^ 0.8728	0.9912	50–10000	50	2.64	3.81

n.d., means not detected in samples.

**Table 4:** The precision and accuracy of the analytes (*n* = 6).

Compound	Expected conc. (ng/mL)	Calculated conc. (ng/mL)	RSD%	Accuracy%
AA-III	5.00	4.99	4.84	99.82
	20.00	20.45	8.12	102.27
	100.00	102.66	3.78	102.66
	750.00	712.39	3.88	94.99

AA-VIIa	5.00	4.81	2.60	96.20
	20.00	19.11	5.97	95.53
	100.00	100.70	2.17	100.70
	750.00	729.75	3.00	97.30

AA-Iva	5.00	4.90	3.43	98.02
	20.00	20.33	3.55	101.67
	100.00	104.33	2.75	104.33
	750.00	703.47	2.77	93.80

AL-I	5.00	5.27	1.65	105.39
	20.00	20.20	4.23	101.00
	100.00	97.13	2.01	97.13
	750.00	721.62	3.63	96.22

AA-II	5.00	5.28	7.88	105.52
	20.00	19.46	6.11	97.32
	100.00	96.61	3.12	96.61
	750.00	760.87	4.96	101.45

AA-I	5.00	5.24	6.47	104.76
	20.00	19.42	3.10	97.08
	100.00	89.78	2.52	89.78
	750.00	782.71	4.73	104.36

Sesamin	50.00	46.80	3.44	93.61
	200.00	206.56	2.98	103.28
	1000.00	1101.68	1.72	110.17
	7500.00	6932.16	2.36	92.43

Asarinin	50.00	45.09	1.92	90.18
	200.00	220.18	2.13	110.09
	1000.00	1121.56	2.12	112.16
	7500.00	6867.46	2.08	91.57

**Table 5:** The recovery result of the analytes (*n* = 3).

Compounds	Recovery-50%		Recovery-100%		Recovery-150%	
	Average (%)	RSD (%)	Average (%)	RSD (%)	Average (%)	RSD (%)
AA-VIIa	85.41	4.78	82.46	5.21	81.58	8.62
AA-IVa	97.74	3.59	97.13	3.89	96.82	5.47
AL-I	109.73	8.40	103.64	9.50	101.10	9.98
AA-I	93.06	5.83	93.87	6.86	96.08	6.76
Sesamin	102.58	5.77	99.53	1.71	96.63	1.71
Asarinin	96.52	8.93	89.43	0.19	85.56	2.31

## Data Availability

The data used to support the findings of this study are available from the corresponding author upon request.

## Abbreviations

AA: Aristolochic acid
AAs: Aristolochic acids derivatives
AL-I: Aristololactam I
AR: Asari radix et rhizoma
HPLC: High-performance liquid chromatography
LOQ: Limit of Quantitation
MRM: Multiple reaction monitoring
RSD: Relative standard deviation
UHPLC-QqQ-MS: Ultra-high-performance liquid chromatography-triple quadrupole tandem mass spectrometry
UHPLC-QTOF/MS: Ultra-high-performance liquid chromatography-time of flight/mass spectrometry.
